# The emerging roles of GPR158 in the regulation of the endocrine system

**DOI:** 10.3389/fcell.2022.1034348

**Published:** 2022-11-18

**Authors:** Jinju Lin, Qin Li, Xiaohua Lei, Huashan Zhao

**Affiliations:** Center for Energy Metabolism and Reproduction, Shenzhen Institute of Advanced Technology, Chinese Academy of Sciences, Shenzhen, China

**Keywords:** GPR158, endocrine, glucocorticoid, prostate, adrenal gland, energy metabolism

## Abstract

G protein-coupled receptor 158 (GPR158) is a member of class C G protein-coupled receptors (GPCRs) and is highly expressed in the central nervous system (CNS) while lowly expressed in peripheral tissues. Previous studies have mainly focused on its functions in the CNS, such as regulating emotions, memory, and cognitive functions, whereas studies on its role in the non-nervous system are limited. It has been recently reported that GPR158 is directly involved in adrenal regulation, suggesting its role in peripheral tissues. Moreover, GPR158 is a stable dimer coupled to the regulator of G protein signaling protein 7 (RGS7) that forms the GPR158-RGS7-Gβ5 complex. Given that the RGS7-Gβ5 complex is implicated in endocrine functions, we speculate that GPR158 might be an active component of the endocrine system. Herein, we reviewed the relevant literature on GPR158, including its molecular structure, regulatory molecules, expression, and functions, and highlighted its roles in endocrine regulation. These findings not only enhance our understanding of GPR158 from an endocrine perspective but also provide valuable insights into drug exploration targeting GPR158 and their applicability in endocrine disorders.

## Introduction

GPR158 was first identified in 2005; Bjarnadóttir et al. searched for genes encoding class C GPCRs in four vertebrates and four invertebrates and classified them into four phylogenetic groups (Group I-IV). GPR158, together with γ-aminobutyric acid (GABA), belongs to Group III (Bjarnadóttir et al., 2005). It plays a pivotal role in modulating physiological function and are targets of several investigational drugs (Ellaithy et al., 2020). To date, the ligand of GPR158 remains unknown, and thus studies on the deorphanization of GPR158 are ongoing (Fu et al., 2022).

According to the Genotype-Tissue Expression database, GPR158 is highly expressed in the CNS, including the cortex, hippocampus, and hypothalamus, while its expression level is lower in peripheral tissues (Obri et al., 2018). Therefore, researchers have predominantly focused on the role of GPR158 in the CNS. Notably, an increasing number of studies indicate that GPR158 is related to endocrine regulation. For instance, glucocorticoid disorders could lead to depression *via* the modulation of GPR158 levels (Sutton et al., 2018). Moreover, the up-regulation of GPR158 in prostate cancer (PCa) and ovarian carcinoma indirectly affects the endocrine system (Patel et al., 2015; Engqvist et al., 2019).

Thus, in this review, literature in PubMed was searched with the keyword “GPR158,” and 43 articles and eight reviews were retrieved. Among them, 15 articles were related to endocrine alterations and the correlation between GPR158 and the endocrine system, such as its structure, regulatory molecules, expression profile, and endocrine-related functions.

## Structural characteristics of GPR158-RGS7-Gβ5 complex

In 2012, Orlandi et al. (2012) reported that GPR158 binds with RGS7 to target the RGS7-Gβ5 complex in the plasma membrane in the CNS. Extracellularly, GPR158 contains a leucine repeat region and a calcium-binding EGF-like domain at the N-terminal and lacks the Venus flytrap module, which is necessary for ligand binding and receptor activation in class C GPCRs. Meanwhile, silico analysis signaled that GPR158 has a signature motif of the metabolic glutamate receptor family at the beginning of the 7th helix, and the N-terminal contains several N-glycosylation sites. Similar to many GPCRs, GPR158 contains cysteine residues at analogous locations between extracellular loop (EL)1 and EL2 that are responsible for ligand recognition (Patel et al., 2013). Moreover, the C-terminal of GPR158 contains three conservative regions similar to other known G protein regulators, which are crucial for binding to RGS7 and enhancing its function in regulating GTPase-activating proteins (Ross and Wilkie, 2000). Knockout of GPR158 in mice led to post-transcriptional destabilization of RGS7 and its inability to localize to the membrane, suggesting that GPR158 is an essential regulator of RGS7 in the CNS and plays a key role in controlling its expression, membrane localization, and catalytic activity (Orlandi et al., 2015). In 2022, Patil et al. (2022) analyzed the near-atomic-level structure of GPR158 *via* single-particle cryo-electron microscopy and described that GPR158 is a homodimeric organization stabilized by phosphatidylinositol and phosphatidylethanolamine. Its N-terminal is the characteristic cache domain fold, an unusual ligand-binding domain in GPCRs. In contrast, its C-terminal directly binds to RGS7, which subsequently binds to Gβ5. This anchoring differs from the classical GPCRs that require the participation of the α subunit of G protein, implying that the GPR158-RGS7-Gβ5 complex may have its unique signaling pathway. Interestingly, RGS7-Gβ5 is also expressed in the corticotroph-derived pituitary AtT-20 cell line, pituitary gland, and pancreatic islets (Nini et al., 2012). A previous study described that overexpression of RGS7-Gβ5 in pancreatic β cells promoted insulin synthesis *via* stimulation of the muscarinic cholinergic receptor M3, indicating that the RGS7-Gβ5 complex plays a decisive role in the endocrine system (Wang et al., 2017).

## Regulatory molecules of GPR158

Although GPR158 is regarded as an orphan receptor, some putative ligands or regulatory molecules have been reported to be associated with it, including polypeptides, intracellular binding proteins, steroid hormones, glycosaminoglycans, and microRNAs (Table 1). Osteocalcin (OCN), as the putative ligand of GPR158, can improve mouse hippocampus-dependent memory (Khrimian et al., 2017), in which the histone-binding protein RbAp48 is an active component (Kosmidis et al., 2018). In nerve growth factor-induced neurite outgrowth of PC12 cells, OCN interacts with GPR158 and promotes PC12 cell proliferation, differentiation, and survival (Ando et al., 2021). Embryonic OCN/GPR158 signals determine lifelong adrenal steroidogenesis and homeostasis in the adrenal gland of mice (Yadav et al., 2022). Indeed, steroid hormones, such as glucocorticoids and androgens, can interact with GPR158. In trabecular meshwork cells (TBM), glucocorticoids can stimulate the expression of GPR158, which promotes proliferation and reduces the permeability of TBM (Patel et al., 2013). Under chronic stress conditions, GPR158 responds to glucocorticoids and induces depression (Sutton et al., 2018). In the early stage of PCa, androgen can upregulate the expression of GPR158 (Patel et al., 2015). Besides the aforementioned hormones, GPC4, as a heparan sulfate proteoglycan (HSPG), interacts with GPR158 in the hippocampal CA3 region and organizes mossy fiber-CA3 synapse density and size (Condomitti et al., 2018). In addition, some microRNAs could also govern the expression of GPR158. In glioma, microRNA-449a down-regulates the expression of GPR158 to regulate neural differentiation and apoptosis (Li et al., 2018). In osteosarcoma, miR613 inhibits the expression of GPR158, leading to the inhibition of the proliferation and angiogenesis of osteosarcoma (Wang et al., 2022). Collectively, the deorphanization of GPR158 still necessitates further investigation in order to identify novel molecules regulating the endocrine system.

**TABLE 1:** Overview of GPR158 regulatory molecules.

Classification	Regulatory molecules	Species/cell types	Location	Function	References
Peptide hormone	OCN	mouse	Hippocampal CA3 region	Improves hippocampal-dependent memory	Khrimian et al. (2017)
		Rat pheochromocytoma cell line PC12	—	Promotes proliferation, differentiation, and survival of PC12 cells	Ando et al. (2021)
		mouse	Adrenal gland	Determines adrenal steroidogenesis and homeostasis^a^	Yadav et al. (2022)
Intracellular binding protein	RGS7	HEK293T/17 cells	membrane	Targets RGS7 to the plasma membrane and augments the activity of GTPase-activating proteins^a^	Orlandi et al. (2012)
	RbAp48	mouse	Hippocampal CA3 region and dentate gyrus	Improves age-related memory loss	Kosmidis et al. (2018)
Steroid hormone	Glucocorticoid	human	Trabecular meshwork cell (TBM)	Promotes proliferation and differentiation of TBM and increases the barrier function of monolayer cells^a^	Patel et al. (2013)
		mouse	Prefrontal cortex	Leads to depression^a^	Sutton et al. (2018)
	Androgen (DHT)	PHPECs and LNCaP cells	—	Stimulates androgen receptor and prostate-specific antigen expression^a^	Patel et al. (2015)
Glycosaminoglycan	Heparan sulfate proteoglycan (HSPG) glypican (GPC4)	HEK293T cells	Hippocampal granule cell axons (mossy fibers)	Organizes mossy fiber-CA3 synapse density and size	Condomitti et al. (2018)
microRNA	microRNA-449a	mouse	glioma	Antagonizes neural differentiation and apoptosis of glioma stem cells	Li et al. (2018)
	miR613	Human osteosarcoma cell lines (U2OS and MG63)	—	Inhibits the proliferation and angiogenesis of osteosarcoma	Wang et al. (2022)

^a^ Indicates association with endocrine function.

## Expression of GPR158 in endocrine-related tissues

*In situ* hybridization and qPCR have revealed that GPR158 is highly expressed in the CA3 region of the hippocampus, cortex, midbrain, brainstem, and cerebellum (Khrimian et al., 2017). Nevertheless, its expression in the hypothalamus has not been explored so far. In 2007, gene expression profiling analysis of the metastatic process of PCa determined the expression of GPR158 in peripheral glands (Chandran et al., 2007). Using affymetrix oligonucleotide arrays to analyze 24 androgen-ablation-resistant metastatic samples and 64 primary prostate tumor samples, the authors found that GPR158 is up-regulated during PCa metastasis. In the female reproductive system, 17 of 29 mucinous ovarian cancer samples (59%) were GPR158-positive (Engqvist et al., 2019). In estrogen-sensitive breast cancer, the expression of GPR158 is down-regulated following the withdrawal of estrogen or the use of estrogen antagonists (Salazar et al., 2011), suggesting that GPR158 may be a novel biomarker for the prognosis of ovarian carcinoma and breast cancer. Recently, a study on OCN regulation of adrenal steroidogenesis in mice revealed that GPR158 is expressed in the adrenal gland, and the expression level is significantly higher than in the hypothalamus and pituitary gland (Yadav et al., 2022). Overall, despite the expression level of GPR158 in peripheral endocrine tissues being lower than that in nervous tissue, this does not influence its physiological role owing to the powerful effects of hormones.

## Endocrine-related functions of GPR158

### GPR158 affects cognition through bone-derived hormone OCN

OCN, as a putative ligand of GPR158, was first discovered by Khrimian et al. (2017) in 2017. By injecting plasma from young mice into aged mice with evident hippocampal-dependent memory impairments, they found that the memory and anxiety-like behaviors were significantly ameliorated. Contrastingly, the absence of this effect in the plasma of OCN knockout (KO) mice confirmed that OCN can improve memory and cognitive abilities. Furthermore, the failure of plasma from OCN KO mice to improve cognitive functions and memory in aged mice was not illustrated by the accumulation of β2-microglobulin, a progeronic molecule in plasma. This phenotype could be reversed by supplementing mouse recombinant uncarboxylated OCN exogenously into the plasma of OCN KO mice. Meanwhile, tissue metalloproteinase inhibitor 2 (TIMP2), a beneficial molecule on cognition in the hippocampus, does not mediate this effect. These results signify that OCN signaling in the brain is independent of β2-microglobulin and TIMP2 and may function through the hormonal pathway. In contrast, GPR158 can interact with OCN in the CA3 region to promote the expression of brain-derived neurotrophic factor (BDNF), thereby improving hippocampal-dependent memory in mice and demonstrating that GPR158 is involved in enhancing memory and cognitive processes. Intracellularly, OCN binds to GPR158 and Gαq on the hippocampal cell membrane, while the amount of pull down of Gαq is significantly decreased in GPR158 KO mice. Furthermore, OCN can upregulate the expression of inositol triphosphate (IP3) but does not affect cAMP expression, indicating that OCN acts through the Gαq-IP3 signaling pathway (Khrimian et al., 2017). Lastly, RbAp48 is required for GPR158/OCN signaling to improve age-related memory decline (Kosmidis et al., 2018).

### GPR158 determines adrenal steroidogenesis through OCN

Glucocorticoid hormones are secreted by cells in the adrenal cortex zona fasciculata, and their synthesis and secretion are regulated by the hypothalamic-pituitary-adrenal axis (Gjerstad et al., 2018). Clinically, glucocorticoids are primarily used to treat various allergic diseases, but excessive use of glucocorticoids can cause numerous side effects, such as osteoporosis (Henneicke et al., 2014). Glucocorticoids inhibit the function of osteoblasts and further attenuate the secretion of OCN. Conversely, OCN enhances the function of glucocorticoids through a classical hormonal feedback loop. GPR158 is expressed in the zona fasciculata and zona globule of the adrenal cortex but not in the adrenal medulla. The binding of embryonic OCN to GPR158 promotes the proliferation of adrenal cells and increases the expression of SF1, a transcription factor essential for adrenal gland development. Embryonic adrenal cells express SF1 and generate Gli1-positive adrenal cortical progenitors in the adrenal capsule, which differentiate into glucocorticoid-producing cells. The hormones released from these cells eventually control blood pressure, blood K+ concentration, and circulating corticosterone levels in acute stress conditions. However, GPR158 KO in the hypothalamus does not affect the expression levels of corticosterone and aldosterone in mice, suggesting that OCN promotes adrenal steroid hormone secretion independently of the hypothalamic-pituitary axis (Yadav et al., 2022).

### GPR158 exacerbates ocular hypertension after glucocorticoid treatment

Ocular hypertension is a severe side effect of glucocorticoid overdose (Dibas and Yorio, 2016). Glucocorticoid drugs, including dexamethasone and triamcinolone acetonide, drive the activity of the GPR158 promoter, thereby increasing its transcription level. In the post-transcription stage, two separate bands (∼70 kDa and ∼55 kDa) can be detected using the C-terminal antibody; likewise, two bands (∼95 -kDa and ∼80-kDa) can be detected using the N-terminal antibody, consistent with those found in prostate cells. This observation indicates that the hydrolysis of GPR158 overexpression occurs in two different locations: EL2 and the C-terminal surrounding the cytosolic tail of the 8th helix. These GPR158 molecular fragments at the C-terminal might participate in GPR158 nuclear entry (Patel et al., 2013). The localization of GPR158 to the nucleus is essential for its function. Following treatment with two different endocytic inhibitors, ConA and CPZ, GPR158 is localized to the plasma membrane. The 8th helical region of GPR158 contains a bipartite nuclear localization signal (NLS), and a different mutant part of NLS in GPR158 would disturb its location in the nucleus. This indicates the necessity of bipartite NLS for GPR158 transfer into the nucleus (Patel et al., 2013). GPR158 enhances TBM cell proliferation by regulating the expression of cyclin D1, a key protein in the transition from the G1 to the S phase of the cell cycle. GPR158 reduces TBM cell monolayer permeability by increasing the expression of tight junction-specific proteins, resulting in the obstruction of aqueous humor backflow and the occurrence of ocular hypertension, which may eventually lead to primary open-angle glaucoma. Glaucoma is treated by lowering the intraocular pressure (IOP). Several different GPCRs can be used as the target of IOP-lowering drugs, such as β-adrenergic antagonists, including atenolol, betaxolol, and carteolo (Toris, 2010). Owing to the pressure regulation of GPR158 in TBM cells, it could be reckoned that inhibition of GPR158 expression may be a new strategy for the treatment of glaucoma.

### GPR158 controls stress-induced depression by responding to glucocorticoid

The medial prefrontal cortex (mPFC) is the region regulating emotion in the brain. Exposure to chronic stress can lead to major depressive disorders (MDD). Glucocorticoids are continuously released during chronic stress conditions, which can lead to the development of maladaptive responses (Myers et al., 2014). Patel et al. (2013) reported that glucocorticoids induce GPR158 expression during chronic stress conditions. Exposure to chronic physical restraint stress subsequently up-regulates GPR158 expression in the glutamatergic neurons of mPFC, thereby leading to depression. Analysis of GPR158 expression in the dorsolateral PFC (dlPFC) of MDD subjects uncovered that GPR158 expression was significantly higher in the dlPFC of MDD patients than that of control subjects, suggesting that GPR158 may regulate the occurrence of depression through neuropathological mechanisms. Deficiency of GPR158 increases the spine density of glutamatergic neurons in the layers of 2/3 of the mPFC and improves expression levels of AMPAR and BDNF, thereby exerting antidepressant effects (Duman and Monteggia, 2006; Autry et al., 2011). GPR158 knockdown also increased the expression of cAMP in the mPFC. As previously mentioned, GPR158 binding to OCN initiates the Gαq-IP3 signaling pathway, which is independent of cAMP (Khrimian et al., 2017). Under chronic stress conditions, glucocorticoid stimulation increases the transcription of GPR158, which links stress, depression, and synaptic plasticity at the molecular level (Sutton et al., 2018), suggesting that GPR158 may be closely related to the neuroendocrine through BDNF.

### GPR158 aggravates PCa progression after stimulation of androgen

The early stage of PCa is androgen-dependent and hence can be treated by androgen deprivation. Nonetheless, in the later stage of cancer, it becomes androgen-independent, also referred to as castration-resistant PCa (Desai et al., 2021). GPR158 overexpression can promote PCa cell proliferation, while endogenous GPR158 siRNA treatment can inhibit cell growth. Administration of the androgen receptor (AR) antagonist bicalutamide inhibited DHT-mediated GPR158 expression in primary human prostate epithelial cells. This also signifies that GPR158 needs to be localized in the nucleus to execute its function. *Pten* is a tumor suppressor gene, and *Pten* KO mice are an accepted PCa animal model. GPR158 is up-regulated in *Pten* KO PCa models co-localized with AR, implying that the expression of GPR158 during androgen deprivation therapy sensitizes PCa to low androgen conditions, thereby accelerating PCa progression (Wang et al., 2003; Patel et al., 2015). The development of castration-resistant PCa includes multiple pathways, including AR co-activation and co-inhibitory protein expression (Shiota et al., 2011). GPR158 co-localizes with AR, illustrating that GPR158 may act as a co-activator of AR and promote the development of PCa. GPR158 expression is associated with a neuroendocrine cell phenotype present in scattered foci of prostate cancer. In LNCaP cells, a human PCa cell line, GPR158 also promoted colony formation, indicating that GPR158 may increase tumorigenicity. Collectively, androgens are necessary to maintain normal physiological functions, but the role of GPR158 in influencing the secretion of AR under physiological conditions remains to be elucidated. In PCa, GPR158 aggravates tumor progression, and its role in regulating other diseases caused by androgen secretion disorders remains unknown.

### GPR158 participates in energy metabolism

GPR158 is involved in the regulation of energy metabolism in both animal models and humans. In 2016, a microarray analysis of mRNAs in the micro-dissected hypothalamic and thalamic control areas yielded 17,745 expression values for RNAs representing protein-coding genes that could be assigned annotated gene names. High relative values of genes are expressed in the medial habenula (MHb), lateral habenula (LHb), and thalamus. Notably, LHb is genetically more closely related to the thalamus than MHb. The study pointed out that GPR158 is up-regulated in the habenular region, suggesting its involvement in regulating food and energy expenditure (Wagner et al., 2016). In 2017, a genome-wide association study using a custom genotyping array found that the GPR158 variant is associated with energy expenditure (EE) in Pima Indians. By analyzing rs11014566, DNA fragments of GPR158, they demonstrated the correlation between GPR158 and EE. GPR158 impacts the obesity genes RGS7 and N-type voltage-gated calcium channel (CACNA1B). Custom genotype analysis of Pima Indians identified a new gene locus in GPR158 that affects EE and predisposition to weight gain. PCR results show that GPR158 is predominantly expressed in the whole brain, hypothalamus, pituitary gland, and liver (Piaggi et al., 2017). Taken together, GPR158 interacts with RGS7 and CACNA1B in the CNS, suggesting that it may influence the body’s food intake and energy metabolism by affecting the hypothalamic-pituitary axis.

## Conclusion

**FIGURE 1:**
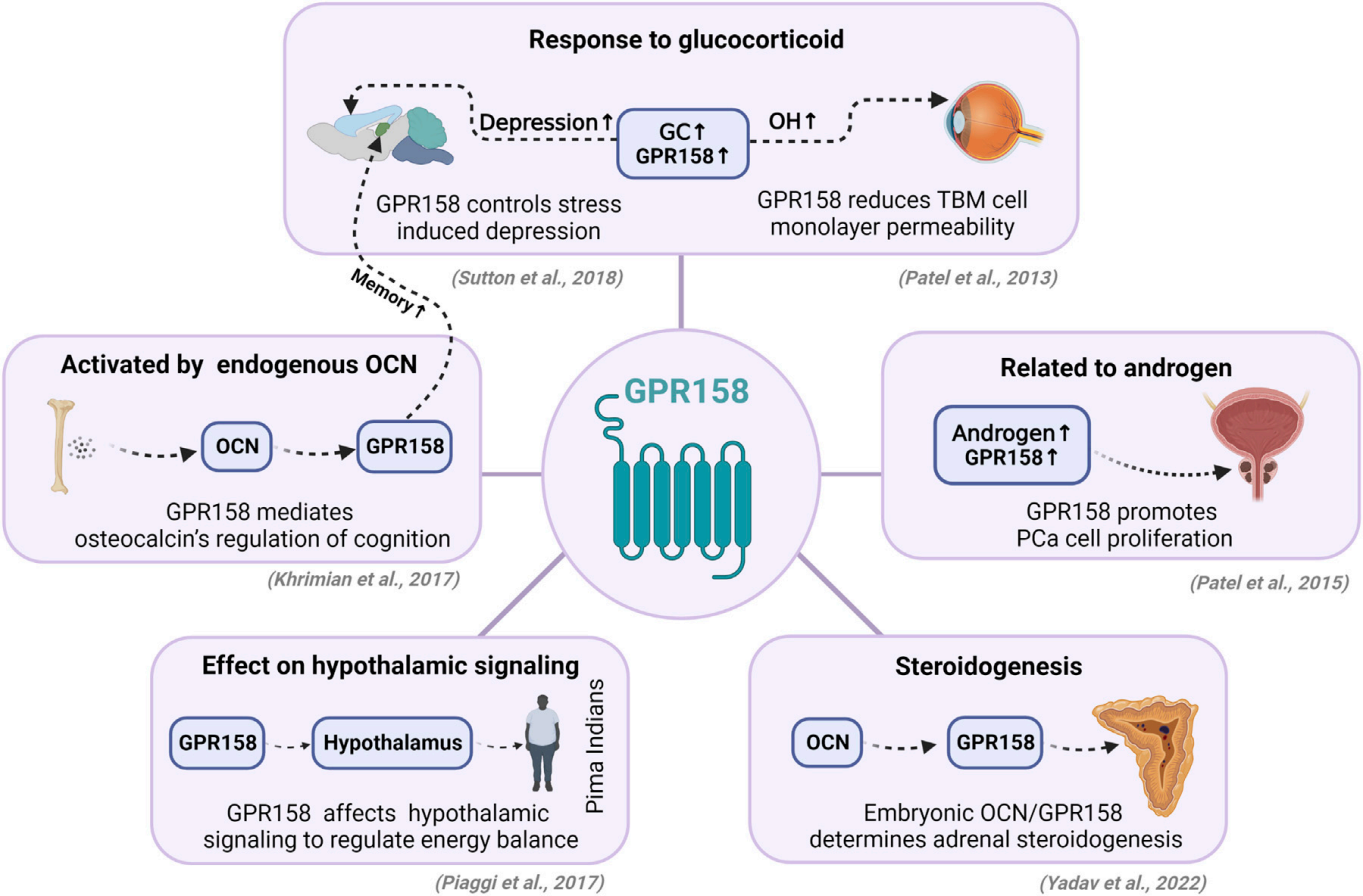
Endocrine-related functions of GPR158. GPR158 is abundantly expressed in the nervous system and participates in the pathogenesis of depression, glaucoma, *etc.*, but some studies indicate that GPR158 is related to endocrine pathways. In the nervous system, GC is directly involved in GPR158-mediated depression and alterations in intraocular pressure. The peripheral bone-derived hormone OCN can affect cognitive functions *via* GPR158. Moreover, a study in Pima Indians confirmed that GPR158 regulates energy balance by affecting hypothalamic signaling. In peripheral tissues, GPR158 is also closely associated with androgen and androgen receptors, thereby regulating the proliferation of prostate cancer cells. Interestingly, the relatively high expression of GPR158 in the adrenal gland can directly affect the generation of adrenocorticosteroids through OCN. GC, glucocorticoid; OH, ocular hypertension; TBM, trabecular meshwork; OCN, osteocalcin; PCa, prostate cancer. GC, glucocorticoid; OH, ocular hypertension; TBM, trabecular meshwork; OCN, osteocalcin; PCa, prostate cancer. The figure was created with BioRender.com.

With the discovery of various regulatory molecules, the functions of GPR158 in peripheral tissue are constantly being updated, especially its role in the endocrine system, despite its low expression levels in the peripheral system (Figure 1). There are still questions to be addressed. For instance, given the expression level of GPR158 in ovarian cancer, GPR158 might have a similar effect through estrogen as that of androgen in PCa. RGS7-Gβ5 is involved in the regulation of the endocrine system, and therefore the endocrine function of the GPR158-RGS7-Gβ5 complex necessitates further exploration. Novel drugs targeting GPR158 showed great promise, but the number of drugs targeting it remains limited. We have established a drug delivery system targeting GPR158 in the brain for either cell labeling or therapeutic purposes (Zhao et al., 2021), which is a preliminary study lacking a thorough exploration of the underlying mechanism. Despite studies on GPR158 being underway, existing studies suggest that GPR158 might be a potential target for endocrine diseases.
